# Developing a framework to guide intervention planning to reduce heat exposure and poor air quality in school classrooms: a scoping review protocol

**DOI:** 10.1136/bmjopen-2025-107367

**Published:** 2025-11-09

**Authors:** Natasha Naidoo, Thandi Kapwata, Muthise Bulani, Shalin Bidassey-Manilal, Poovie Reddy, Melishnee Ruthanam, Caradee Yael Wright

**Affiliations:** 1 Environment and Health Research Unit, South African Medical Research Council, Durban, South Africa; 2 Environment and Health Research Unit, South African Medical Research Council, Pretoria, Gauteng, South Africa; 3 Environmental Health, University of Johannesburg, Doornfontein, South Africa; 4 Department of Community Health Studies, Environmental Health, Durban University of Technology, Durban, South Africa

**Keywords:** Climate Change, Schools, Community child health, Health, Health Equity

## Abstract

**Abstract:**

**Introduction:**

Poor indoor air quality and heat, individually and together, cause serious health impacts on children. Thus, there is a growing interest in creating school classroom environments that reduce health risks associated with these indoor environmental conditions. However, it is unclear if the existing evidence provides effective, practical and reliable interventions or strategies that can be implemented in classrooms. Additionally, the pertinence of these strategies for low-income communities needs to be elucidated. This scoping review will, therefore, document the findings of studies that have analysed interventions and strategies to improve school classroom conditions by reducing heat exposure and poor air quality to protect the health and well-being of children. This scoping review will consider: (1) interventions or adaptation strategies that have reduced heat exposure in classrooms; (2) interventions or adaptation strategies that have reduced air pollutant exposure in classrooms; (3) classroom building modifications that reduce exposure to heat and poor air quality and (4) improved health outcomes in children due to reduced heat and air pollutants. Studies that report reductions in heat or air pollutant exposure and show significant improvements in learner health outcomes will be prioritised for deeper analysis and considered particularly valuable for informing evidence-based recommendations.

**Methods and analysis:**

We will explore original and review articles from both high-income and low-income settings that evaluate interventions and strategies for preventing or reducing heat exposure and poor air quality, to safeguard the health and well-being of children in classrooms. A comprehensive literature search will be conducted on Ovid MEDLINE, Ovid Global Health, PubMed, Scopus, ScienceDirect and Web of Science. Searches will be limited to literature published in the last 10 years (2015–2025). Results will be exported to EndNote for deduplication and to Abstrackr software for screening. Four reviewers will do abstract screening to ensure consistency. Data from included papers will be presented in tables with a narrative commentary.

**Ethics and dissemination:**

No ethical approval is required for this study as primary data collection will not be conducted. A manuscript detailing the findings from this review will be published in a peer-reviewed journal.

**STRENGTHS AND LIMITATIONS OF THIS STUDY**

- This scoping review will provide us with a broad perspective of the interventions and strategies that have been published in internationally recognised peer-reviewed literature for preventing or reducing heat and poor air quality in classrooms around the world.
- The review will also discuss the most cost-effective, accessible and effective strategies that can be easily implemented in low-income communities.
- The strength of the study is that it could provide innovative and sustainable strategies from developed countries that may be modified for use in low-income communities.
- A potential limitation of this study is that there may be few qualifying studies that meet the criteria of exploring interventions/strategies than those that investigate the health impacts of heat and poor air quality.
- A limitation of this study is that the quality of the evidence will not be assessed.

## Introduction

 Governments and policy-makers require practical and accessible strategies that prevent adverse health effects from poor indoor environmental conditions.^1^ This is especially important for populations who are more susceptible to health impacts from indoor heat and poor air quality, such as children.^2 3^ Due to their developing immune and thermoregulatory systems, children are more vulnerable to adverse health effects.^4^ Children are considerably smaller, with lower body weight than adults and a proportionally greater body surface area that makes them more vulnerable to higher temperatures.^4^ Additionally, small children breathe in air at a more rapid pace than adults.^5^ Taken together, these physical characteristics render children more sensitive to environmental exposures like heat and air pollution.^4 5^ Exposing children to indoor heat and poor air quality may worsen impacts on their health and wellness.

In temperate climates, such as countries in Europe, children are susceptible to heat-related stress as they have a lower propensity to acclimatise to high temperatures, which occur during summer months.^6^ For example, young children under 5 years of age, confined to vehicles, are susceptible to death during heatwaves and heat stroke in playgrounds without shading.^7^ In England, primary school children experience cognitive decline on hot days and prefer temperatures of 22°C–24°C, with increasing discomfort above 25°C.^8^ The CLIMate ADAptation (CLIMADA) spatial risk assessment platform assessed overheating risk at ~20 000 schools in England.^9^ The predictive model showed that as global temperatures rise, there may be a significant increase in the risk of classroom overheating with up to 15 days per year exceeding 35 °C and becoming more frequent than those just above 26°C.^9^ Models such as the CLIMADA spatial risk assessment model can be used in any setting to map the heat index of school classrooms to classroom characteristics and inform policy-makers of school characteristics that may be advantageous to decrease the thermal heat indices.^9^

In low-income countries, climate change-related impacts are often magnified due to a combination of poor infrastructure and limited resources for adaptation and resilience to heat and air pollutants.^10^ In African, South Asian and Latin American countries, schools often lack proper ventilation, insulation and cooling mechanisms, exacerbating childhood exposure to both heat stress and indoor air pollution.^11^ In Nigeria, students favour short-walled, open-plan buildings with natural ventilation over enclosed classrooms.^12^ Open-plan classrooms are deemed necessary to save on air-conditioning, which is deemed expensive and energy-intensive.^13^ In enclosed classrooms in an urban industrial area in Cameroon where temperatures were >32°C, the majority of students felt extremely hot (48%), were fatigued (76%) and experienced headaches (38%).^14^ Headaches were more common from noon to afternoon when temperatures were at their highest (32.5°C–36.6°C).^15^ In one study in South Africa, schools in the lower income brackets were enclosed but the buildings lacked wall and ceiling insulation that may enhance consistently lower indoor temperatures.^16^ As a result of inadequate building insulation, rising ambient (outdoor) temperatures lead to increased inside temperatures. The compounding effects of environmental stressors pose both short-term and long-term health risks, such as acute and chronic respiratory conditions and increased cases of heat-related illnesses.^11^ This context underscores the urgent need for interventions to mitigate these environmental hazards, particularly in low-to-middle-income countries (LMICs), where the consequences of inaction are profound.

Prevention and response measures to reduce indoor heat and poor air quality can safeguard children during their time spent in classrooms and are essential for their overall health and development.^15^ In low-income communities, vulnerable populations, especially children, are disproportionately affected by high indoor temperatures and poor air quality due to inadequate infrastructure, limited access to cooling solutions and high levels of air pollution.^14^ Despite substantial evidence on the health risks of heat and poor air quality in classrooms, limited effort exists to regulate these environmental factors and mitigate adverse health outcomes in children. A potential challenge might be that the available evidence in the literature is neither clear nor accessible enough to inform better policies for the improvement of classroom environments.

This scoping review aims to identify and assess strategies that effectively reduce the negative health impacts of heat exposure and poor air quality in classrooms. By examining original studies that focus on sustainable solutions from both high-income and low-income settings, this review will highlight the available evidence that demonstrates practical and accessible strategies that help prevent health risks by reducing indoor heat and poor air quality in classrooms. A guiding framework will be developed that illustrates the practicality, accessibility, affordability and sustainability of the interventions and strategies chosen from the literature and will be useful to decision-makers. Decision-makers across government and civil society in all settings will be able to plan for the steadily rising temperatures and poor air quality experienced by their populations and establish relevant guidelines for all settings where children attend schools. In addition, these innovative, sustainable, practical and accessible guidelines will benefit low-income communities in semidry, arid tropical and subtropical regions, whose populations are disproportionately affected.

### Aim and objectives

To gather the available studies that have provided solutions to improve school classroom conditions by reducing heat exposure and poor air quality and safeguarding the health and well-being of children. In addition, we will identify the most effective strategies for climate-proofing classrooms in low-income communities that are practical, accessible, affordable and sustainable.

The objectives of this review are to

- To gather evidence on strategies/interventions designed to reduce indoor heat and poor air quality in classrooms.
- To identify building characteristics and environmental factors (such as tree shading or insulation) that influence temperatures in classrooms.
- To highlight gaps in health research related to heat and poor air quality in various climatic zones and regions.
- To compare strategies from both high-income and low-income settings, identifying those that have demonstrated effectiveness in reducing health risks.
- To formulate a framework/model of change that proposes practical, scalable and context-specific strategies/interventions that can inform future climate policies for schools.
- To recommend areas for future research, particularly around climate adaptation, resilience and infrastructure development in classrooms.

## Methods and analysis

This scoping review is planned to begin in January 2026. Database searches are expected to be completed by February 2026, with screening, data charting and analysis taking place from March to April 2026. The review is anticipated to be completed by May 2026. To map the available evidence on interventions and strategies aimed at preventing or reducing heat and poor air quality in school classrooms, and to identify gaps for future research and policy development, we will adopt the scoping review frameworks proposed by Arksey and O’Malley^17^ and further refined by Levac *et al*,^18^ alongside the Joanna Briggs Institute method. The review process will include the following key steps: defining objectives and research questions, developing a comprehensive search strategy, establishing inclusion and exclusion criteria, extracting and charting relevant data, and synthesising and presenting the findings as a framework that will help policy development for low-income communities. We will also discuss the implications of the results for policy and future research. To ensure transparency and reproducibility, the review will follow the PRISMA-ScR (Preferred Reporting Items for Systematic Reviews and Meta-Analyses Extension for Scoping Reviews) guidelines (online supplemental table S1).^12^

### Definitions

Low-income community: areas with systemic inequalities, limited resources and disproportionate exposure to environmental hazards. These challenges are not simply the result of individual failings but rather the product of systemic inequalities that perpetuate poverty and marginalisation.^13^

Intervention: any activity aimed at improving human health by preventing disease, treating existing conditions or restoring lost function. It can involve a range of actions, from individual behaviours to community-level programmes, and may target various levels of the social and environmental determinants of health.^16^

### Eligibility criteria

We used the Sample, Phenomenon of Interest, Design, Evaluation, and Research type (SPIDER) framework, which can be applied to environmental health questions, especially if the focus is on exposures and outcomes without requiring a strict comparison group.^19^ In this study, SPIDER provided a straightforward, yet broad path to frame our research focus as follows:

#### Sample

Children or adolescents under 18 years old in school settings.

#### Phenomenon of interest

The impact of adaptations or interventions in response to extreme heat or hot weather and/or poor air quality in classrooms.

#### Design

Studies should be original research publications, from both high-income and low-income countries, with any type of study design that addresses the impact of an adaptation strategy or intervention.

##### Evaluation

Improvements in thermal comfort, reductions in heat-related and air quality-related health issues and/or better cognitive/learning outcomes that will be useful for LMICs.

### Research type

Can be qualitative, quantitative or mixed study types.

### Search strategy

The final search strategy comprises search terms related to heat/air quality, schools, interventions/strategies and child health and an example of a search strategy for Science Direct is given in table 1. Six electronic literature databases (Ovid MEDLINE, Ovid Global Health, PubMed, Scopus, ScienceDirect and Web of Science) will be searched for relevant articles and only English articles will be included. The search will be limited to the last ten years (2015–2025) to ensure that the information is current. The search string will be adapted to the specific technical requirements of each database and an example literature search for the six databases is given in online supplemental table S2.

**Table 1:** Search strategy that will be used to find peer-reviewed publications on Science Direct (for the search strategies of all databases, please refer to online supplemental table S2)

Database	Air quality	Heat exposure
Science Direct	“air quality” OR “air pollution"school* AND (classroom* OR environment* OR building*)indoor AND (“air quality” OR pollut*)ventilat* AND (school* OR classroom* OR build*)health AND (impact* OR effect* OR outcome*)respiratory AND (diseas* OR condition* OR symptom*)cardiovascular AND (diseas* OR risk* OR condition*)mental AND (health OR condition*)child* OR student* OR pupil*	heat AND (expos* OR wave* OR stress*)extreme AND (heat OR temperature)school* AND (classroom* OR environment* OR building*)thermal AND (comfort OR environment)health AND (impact* OR effect* OR outcome*)respiratory AND (diseas* OR condition* OR symptom*)mental AND (health OR condition*)child* OR student* OR pupil*

### Study selection

#### Title and abstract screening

The screening process will be compatible with the PRISMA-ScR standards recommended for peer-reviewed publications. The publications from searches on the respective databases will be imported into EndNote for deduplication. The deduplicated data will be imported into the AbstrackR open-source software. Screening will be done by two authors independently and the inclusion and exclusion criteria for screening are detailed in table 2. Even though the AbstrackR software lacks automated functions for screening, it allows users to focus on data extraction and management and makes it easier for authors. The data charting tables will be constructed in AbstrackR and the resultant table (online supplemental table S3) will be exported to Microsoft Excel (Microsoft 365).

**Table 2:** The inclusion and exclusion criteria used to screen literature

Inclusion criteria	Exclusion criteria
Studies involving children or adolescents 18 years old and under	Studies focused on adults or populations older than 18 years.
Studies conducted in classrooms in educational institutions/school settings, including primary and secondary schools	Studies not conducted in classrooms in school settings (eg, studies outdoors, in homes or other environments).
Studies examining exposure to extreme heat or hot weather conditions in classrooms.	Studies that do not address exposure to heat, hot weather or air quality in classrooms.
Studies assessing poor air quality in classrooms.	Studies focusing on factors unrelated to thermal comfort or air quality (eg, noise pollution, lighting).
Studies that evaluate both heat and air quality in combination or separately.	Literature published more than 10 years ago, unless evidence is highly relevant to the topic.
Studies assessing interventions or adaptations aimed at improving health outcomes/health impacts/health risks/ thermal comfort and/or air quality.	Studies without a focus on interventions or adaptations to improve health outcomes/health impacts/health risks/ thermal comfort or air quality (eg, studies on general health without intervention focus).
Studies that involve modifications to the built environment (eg, classroom ventilation, shading, insulation) or behavioural interventions (eg, adjusting school hours).	Studies that do not provide detailed descriptions or evaluations of adaptations or interventions.
Observational studies, experimental studies or qualitative studies assessing the impact of adaptations or interventions. Original research studies (qualitative, quantitative or mixed methods). Observational or experimental studies describing or evaluating effective adaptations/interventions.	Reviews, editorials or commentaries that do not provide original research data. Theoretical papers, literature reviews or non-original studies (eg, systematic reviews without new data). Grey literature
Studies reporting outcomes such as improvements in thermal comfort, reductions in heat-related or air quality-related health issues (eg, heat stress, respiratory issues).	Studies that do not report on thermal comfort, health outcomes or cognitive/learning outcomes.
Studies that examine cognitive or learning outcomes linked to improvements in thermal comfort or air quality.	Studies that only mention outcomes tangentially related to heat or air quality without focusing on the effects in classrooms.
Studies available in English.	Articles not available in English.

### Full-text screen

The full-text screen will be completed independently by two reviewers (NN, MB and/or SB-M) using the same inclusion and exclusion criteria outlined above. Unlike the title and abstract screen, no software will be used to conduct the screening and monitor agreement between the reviewers’ assessments. However, there will be meetings to determine the applicability of the full-text articles. All differences in screening will be resolved through discussion and consensus with CYW and TK. The reference lists of articles meeting the criteria for full-text review will be manually searched for additional articles relevant to the review.

### Data charting and synthesis

The following data will be extracted from all included studies: first author surname, initial and year, location of study, study design, school type, intervention description, cost-effectiveness, sustainability score, reported barriers of the intervention, equity and transferability to low-income communities. Data extraction will be completed by one reviewer (NN) and peer reviewed by a second (MB). Interpretation of the data will involve analysing the scoring in terms of cost-effectiveness, sustainability, equity and transferability (table 3). In addition, data synthesis will involve analysing the findings from identified studies using thematic analysis.

**Table 3:** Scoring of the interventions and strategies for sustainability, equity and transferability to low-income communities

Sustainability	Cost-effectiveness	Equity	Transferability	Criteria	Score
Imported technology with no maintenance	No mention of costing	Implemented by private or high-income schools	Significant investment needed as it is a high technology solution	Not addressed	0
Imported technology that can be maintained locally	Vague mention of costing	Accessible to all schools regardless of economic status	It is difficult but it is possible to get assistance for procurement of technology	Addressed but limited	1
Enhancement or improvement of existing technology	Moderate but formal economic evaluation		May be implemented with some government/ non-governmental organisation (NGO) assistance	Moderately addressed	2
Low energy or passive technology with long lifespan and local materials	Full cost-effectiveness with affordability matching achieved outcomes		Requires minimal infrastructure, adaptable and community-driven	Well addressed with clear evidence	3

### Framework development

We will develop a conceptual framework to assess interventions aimed at reducing classroom heat and air pollution exposure based on four key criteria: cost-effectiveness, sustainability, equity and transferability to low-income settings. This framework will guide data extraction and synthesis across included studies. Specifically, we will examine how interventions report on or incorporate economic evaluations, long-term viability, accessibility for underserved populations and feasibility in low-resource environments. It should be noted that within the framework, the improvement of health outcomes in children is paramount and will be the main thread running through the interventions and strategies that make the final list. However, improving child health is a long-term goal, and short-term interventions often do not allow enough time to demonstrate measurable impact. As such, we will likely include short-term and medium-term indicators that reflect progress towards improved child health, while encouraging strategies that support ongoing evaluation beyond the immediate implementation phase, thereby emphasising the need to work towards long-term goals.

### Patient and public involvement

There will be no patient or public involvement in this research.

## Ethics and dissemination

As this review involves a synthesis and presentation of available resources, it does not require ethics approval. Results will be published in a peer-reviewed journal, developed into easily disseminated infographics and shared at international conferences.

### Discussion

A proper characterisation of heat-related health adaptations and interventions in classrooms, particularly within low-income communities, is a crucial area for development. It would be highly beneficial to see studies that evaluate the effectiveness of interventions and strategies, not only in response to environmental factors like heat and air quality, but also across a comprehensive range of health outcomes and among diverse populations.

A literature profile of the types of interventions assessed, and the contexts (settings and populations) studied or overlooked within the current climate change and health literature would help inform evidence-based recommendations for practice, policy and research globally, but more importantly identify interventions suitable to low-income communities. Thus, a key strength of our study is the scoring of the interventions and strategies to guide a framework that takes into account equity, sustainability and transferability to low-income communities. There are a number of limitations to this review. The terminology around climate change and extreme weather events, which are the key concepts in this review, is continuously evolving. It is likely that newly emerging terms are not encompassed in our search strategy, which may result in relevant literature being missed. Furthermore, although we selected six databases based on their relevance and coverage of the topic of interest, there may be peer-reviewed articles not indexed by these databases that are not identified. However, we will manually search the reference lists of included articles to identify any additional literature relevant to our review. The geographical orientation of classrooms and the presence/absence of insulation may be underexplored in the available literature. The data on the impact of climatic zones beyond urban and rural areas (eg, mountains, coastal regions) might be limited. The seasonal variations (particularly summer) may not be adequately represented in the research, leading to potential underreporting of disparities in thermal comfort. There may be gaps in literature on long-term health impacts. The exclusion of non-peer- reviewed literature, for example, grey literature, may miss important interventions and strategies that may not have been published in the scientific literature. The potential for not covering all adaptation strategies as some may be in the experimental stages.

## Supplementary material

10.1136/bmjopen-2025-107367 online supplemental table 1

10.1136/bmjopen-2025-107367 online supplemental table 2

10.1136/bmjopen-2025-107367 online supplemental table 3
