# Supplementary material for: Developing a framework to guide intervention planning to reduce heat exposure and poor air quality in school classrooms: a scoping review protocol
Source: BMJ Open. 2025 Nov 9;15(11):e107367. doi: 10.1136/bmjopen-2025-107367 (PMC12598995; doi:10.1136/bmjopen-2025-107367)
Supplement: online supplemental table 2 [file bmjopen-15-11-s002.docx]

**Table S2. Literature search strategies on the six databases chosen for the study**

| **Database** | **Air quality** | **Heat exposure** |
| --- | --- | --- |
| Ovid MEDLINE and Ovid Global Health | 1. *exp* Air Pollution/ or (air adj3 (qualit* or pollut*)).tw,kf. 2. *exp* Schools/ or (school* adj3 (classroom* or environment* or building*)).tw,kf. 3. *exp* Indoor Air Pollution/ or (indoor adj3 (pollut* or air qualit*)).tw,kf. 4. *exp* Ventilation/ or (ventilat* adj3 (school* or classroom* or build*)).tw,kf. 5. *exp* Health Impact/ or (health adj3 (impact* or effect* or outcome*)).tw,kf. 6. *exp* Respiratory Diseases/ or (respiratory adj3 (diseas* or condition* or symptom*)).tw,kf. 7. *exp* Cardiovascular Diseases/ or (cardiovascular adj3 (diseas* or risk* or condition*)).tw,kf. 8. *exp* Mental Health/ or (mental adj3 (health or condition*)).tw,kf. 9. *exp* Children/ or (child* or student* or pupil*).tw,kf. 10. 1 or 2 or 3 or 4 11. 5 or 6 or 7 or 8 12. 9 and 10 and 11 | 1. *exp* Heat Exposure/ or (heat adj3 (expos* or wave* or stress*)).tw,kf. 2. *exp* Extreme Heat/ or (extreme adj3 (heat or temperature)).tw,kf. 3. *exp* Schools/ or (school* adj3 (classroom* or environment* or building*)).tw,kf. 4. *exp* Thermal Comfort/ or (thermal adj3 (comfort or environment)).tw,kf. 5. *exp* Health Impact/ or (health adj3 (impact* or effect* or outcome*)).tw,kf. 6. *exp* Respiratory Diseases/ or (respiratory adj3 (diseas* or condition* or symptom*)).tw,kf. 7. *exp* Mental Health/ or (mental adj3 (health or condition*)).tw,kf. 8. *exp* Children/ or (child* or student* or pupil*).tw,kf. 9. 1 or 2 or 3 or 4 10. 5 or 6 or 7 or 8 11. 9 and 10 and 11 |
| PubMed | (air[tiab] AND (quality[tiab] OR pollution[tiab])) OR "Air Pollution"[MeSH] (school*[tiab] AND (classroom*[tiab] OR environment*[tiab] OR building*[tiab])) OR "Schools"[MeSH] (indoor[tiab] AND (pollut*[tiab] OR "air quality"[tiab])) OR "Indoor Air Pollution"[MeSH] (ventilat*[tiab] AND (school*[tiab] OR classroom*[tiab] OR build*[tiab])) OR "Ventilation"[MeSH] ("health impact"[tiab] OR "health outcome"[tiab] OR "health effect"[tiab]) OR "Health Status"[MeSH] ("Respiratory Tract Diseases"[MeSH] OR respiratory[tiab] AND (disease*[tiab] OR condition*[tiab] OR symptom*[tiab])) ("Cardiovascular Diseases"[MeSH] OR cardiovascular[tiab] AND (disease*[tiab] OR risk*[tiab] OR condition*[tiab])) ("Mental Health"[MeSH] OR mental[tiab] AND (health OR condition*)) (child*[tiab] OR student*[tiab] OR pupil*[tiab]) OR "Child"[MeSH] | (heat[tiab] AND (expos*[tiab] OR wave*[tiab] OR stress*[tiab])) OR "Heat"[MeSH] (extreme[tiab] AND (heat[tiab] OR temperature[tiab])) (school*[tiab] AND (classroom*[tiab] OR environment*[tiab] OR building*[tiab])) OR "Schools"[MeSH] ("thermal comfort"[tiab] OR "thermal environment"[tiab]) OR "Temperature"[MeSH] ("health impact"[tiab] OR "health outcome"[tiab] OR "health effect"[tiab]) OR "Health Status"[MeSH] ("Respiratory Tract Diseases"[MeSH] OR respiratory[tiab] AND (disease*[tiab] OR condition*[tiab] OR symptom*[tiab])) ("Mental Health"[MeSH] OR mental[tiab] AND (health OR condition*)) (child*[tiab] OR student*[tiab] OR pupil*[tiab]) OR "Child"[MeSH] |
| Scopus | TITLE-ABS-KEY(air W/3 (quality OR pollut*)) TITLE-ABS-KEY(school* W/3 (classroom* OR environment* OR building*)) TITLE-ABS-KEY(indoor W/3 (pollut* OR "air quality")) TITLE-ABS-KEY(ventilat* W/3 (school* OR classroom* OR build*)) TITLE-ABS-KEY(health W/3 (impact* OR effect* OR outcome*)) TITLE-ABS-KEY(respiratory W/3 (diseas* OR condition* OR symptom*)) TITLE-ABS-KEY(cardiovascular W/3 (diseas* OR risk* OR condition*)) TITLE-ABS-KEY(mental W/3 (health OR condition*)) TITLE-ABS-KEY(child* OR student* OR pupil*) | TITLE-ABS-KEY(heat W/3 (expos* OR wave* OR stress*)) TITLE-ABS-KEY(extreme W/3 (heat OR temperature)) TITLE-ABS-KEY(school* W/3 (classroom* OR environment* OR building*)) TITLE-ABS-KEY(thermal W/3 (comfort OR environment)) TITLE-ABS-KEY(health W/3 (impact* OR effect* OR outcome*)) TITLE-ABS-KEY(respiratory W/3 (diseas* OR condition* OR symptom*)) TITLE-ABS-KEY(mental W/3 (health OR condition*)) TITLE-ABS-KEY(child* OR student* OR pupil*) |
| Science Direct | "air quality" OR "air pollution" school* AND (classroom* OR environment* OR building*) indoor AND ("air quality" OR pollut*) ventilat* AND (school* OR classroom* OR build*) health AND (impact* OR effect* OR outcome*) respiratory AND (diseas* OR condition* OR symptom*) cardiovascular AND (diseas* OR risk* OR condition*) mental AND (health OR condition*) child* OR student* OR pupil* | heat AND (expos* OR wave* OR stress*) extreme AND (heat OR temperature) school* AND (classroom* OR environment* OR building*) thermal AND (comfort OR environment) health AND (impact* OR effect* OR outcome*) respiratory AND (diseas* OR condition* OR symptom*) mental AND (health OR condition*) child* OR student* OR pupil* |
| Web of Science | TS=(air NEAR/3 (quality OR pollut*)) TS=(school* NEAR/3 (classroom* OR environment* OR building*)) TS=(indoor NEAR/3 (pollut* OR "air quality")) TS=(ventilat* NEAR/3 (school* OR classroom* OR build*)) TS=(health NEAR/3 (impact* OR effect* OR outcome*)) TS=(respiratory NEAR/3 (diseas* OR condition* OR symptom*)) TS=(cardiovascular NEAR/3 (diseas* OR risk* OR condition*)) TS=(mental NEAR/3 (health OR condition*)) TS=(child* OR student* OR pupil*) | TS=(heat NEAR/3 (expos* OR wave* OR stress*)) TS=(extreme NEAR/3 (heat OR temperature)) TS=(school* NEAR/3 (classroom* OR environment* OR building*)) TS=(thermal NEAR/3 (comfort OR environment)) TS=(health NEAR/3 (impact* OR effect* OR outcome*)) TS=(respiratory NEAR/3 (diseas* OR condition* OR symptom*)) TS=(mental NEAR/3 (health OR condition*)) TS=(child* OR student* OR pupil*) |
