# Supplementary material for: Developing a framework to guide intervention planning to reduce heat exposure and poor air quality in school classrooms: a scoping review protocol
Source: BMJ Open. 2025 Nov 9;15(11):e107367. doi: 10.1136/bmjopen-2025-107367 (PMC12598995; doi:10.1136/bmjopen-2025-107367)
Supplement: online supplemental table 3 [file bmjopen-15-11-s003.docx]

Table X. The interventions found in response to heat

| First author. year | Location where the study was conducted | Study design | School type/  educational level | Intervention description | Cost effectiveness | Sustainability score | Reported barriers | Equity | Transferability to low-income community score |
| --- | --- | --- | --- | --- | --- | --- | --- | --- | --- |
|  |  |  |  |  |  |  |  |  |  |
|  |  |  |  |  |  |  |  |  |  |
|  |  |  |  |  |  |  |  |  |  |
|  |  |  |  |  |  |  |  |  |  |

Table X. The interventions found in response to poor air quality

| First author. year | Location where the study was conducted | Study design | School type/  educational level | Intervention description | Cost effectiveness | Sustainability score | Reported barriers | Equity | Transferability to low-income community score |
| --- | --- | --- | --- | --- | --- | --- | --- | --- | --- |
|  |  |  |  |  |  |  |  |  |  |
|  |  |  |  |  |  |  |  |  |  |
|  |  |  |  |  |  |  |  |  |  |
|  |  |  |  |  |  |  |  |  |  |
