# Supplementary material for: Developing a framework to guide intervention planning to reduce heat exposure and poor air quality in school classrooms: a scoping review protocol
Source: BMJ Open. 2025 Nov 9;15(11):e107367. doi: 10.1136/bmjopen-2025-107367 (PMC12598995; doi:10.1136/bmjopen-2025-107367)
Supplement: Supplementary file 4 [file bmjopen-2025-107367.reviewer_comments.pdf]

PEER REVIEW HISTORY

BMJ Open publishes all reviews undertaken for accepted manuscripts. Reviewers are asked to complete a checklist review form and are provided with free text boxes to elaborate on their assessment. These free text comments are reproduced below.

ARTICLE DETAILS

Title (Provisional)

Developing a framework to guide intervention planning to reduce heat exposure and poor air quality in school classrooms: a scoping review protocol

Authors

Naidoo, Natasha; Kapwata, Thandi; Bulani, Muthise; Bidassey-Manilal, Shalin; Reddy, Poovie; Ruthanam, Melishnee; Wright, Caradee Yael

---

VERSION 1 - REVIEW

**Reviewer**	**1**
**Name**	**Perry, Lucy**
**Affiliation**	**Swansea University - Singleton Park Campus, Data science**
**Date**	**20-Aug-2025**
**COI**	

Overall, this is a good protocol with clear rationale and aims, intending to map out recent literature on heat and poor air quality exposure in classrooms.

There are a couple of small points that require clarity.

1. In the abstract methods section and again on page 8, line 187, the authors state that the search will only include literature from the past 10 years. However, within the exclusion table, authors state: "Literature published more than 20 years ago is considered unless highly relevant to the topic". Your meaning is confusing here and needs rewording.

2. Table 1 presents and is titled terms used to find peer-reviewed and grey literature. Yet in your discussion of limitations at the end of the paper, it states that the exclusion of grey literature is a limitation. It would be best to also add this to your inclusion/ exclusion table and remove it from the table title

3. Table 1 does not refer to air quality and poorly explains what these groups are. I feel your supplementary Table 2 is much clearer with your search strategy

---

VERSION 1 - AUTHOR RESPONSE

Reviewer: 1, Miss Lucy Perry, Swansea University - Singleton Park Campus:
Comments to the Author:
Overall, this is a good protocol with clear rationale and aims, intending to map out recent literature on heat and poor air quality exposure in classrooms.

There are a couple of small points that require clarity.

1. In the abstract methods section and again on page 8, line 187, the authors state that the search will only include literature from the past 10 years. However, within the exclusion table, authors state: "Literature published more than 20 years ago is considered unless highly relevant to the topic". Your meaning is confusing here and needs rewording.

Response: We sincerely apologise for this oversight and typing error. We have corrected the phrasing in the exclusion column to reflect what we meant as follows: “Literature published more than 10 years ago, unless evidence is highly relevant to the topic.”

2. Table 1 presents and is titled terms used to find peer-reviewed and grey literature. Yet in your discussion of limitations at the end of the paper, it states that the exclusion of grey literature is a limitation. It would be best to also add this to your inclusion/ exclusion table and remove it from the table title.
Response: We thank the reviewer for their keen observation and we apologize for this error. We have removed reference to grey literature in Table 1 and we have added it to the exclusion criteria in Table 2.

Whilst correcting this error, we noticed that our tables are not cited in the text before they appear. We have now corrected this and cited all our tables in our text, indicated by track changes. We sincerely apologise for this oversight.

3. Table 1 does not refer to air quality and poorly explains what these groups are. I feel your supplementary Table 2 is much clearer with your search strategy

Response: We agree with the reviewer that Table 1 is poorly explained and that the supplementary table is much easier to understand. We thought that the search strategies outlined in the supplementary table may be too long and confusing for the reader of the article, so we attempted to simplify it using Table 1, which may be inadequate and confusing. Thus, we have amended Table 1 to reflect an example of the search strategy from Science Direct and the reader is then referred to Supplementary table 2 for the full complement of search strategies that will be used on all the databases. We decided not to display all search strategies in the main text as it would be a large, untidy table in the main

text that may distract readers from the main content. We humbly hope that the reviewer and editor agree with this direction.

Reviewer: 1

If you have selected ‘Yes’ above, please provide details of any competing interests.: Not applicable
